# Molecular Network-Based Identification of Competing Endogenous RNAs in Thyroid Carcinoma

**DOI:** 10.3390/genes9010044

**Published:** 2018-01-19

**Authors:** Minjia Lu, Xingyu Xu, Baohang Xi, Qi Dai, Chenli Li, Li Su, Xiaonan Zhou, Min Tang, Yuhua Yao, Jialiang Yang

**Affiliations:** 1 College of Life Sciences, Zhejiang Sci-Tech University, Hangzhou 310018, China; luminjia1120@163.com (M.L.); xingyuxu821@163.com (X.X.); xibaohang@sina.com (B.X.); daiailiu2004@aliyun.com (Q.D.); 2 School of Mathematics and Statistics, Hainan Normal University, Haikou 570100, China; lcl904624834@live.com (C.L.); andy_suran@sina.com (L.S.); 3 Institute of Basic Medical Sciences, Wannan Medical College, Hefei 241000, China; zhouxnan820@163.com; 4 Department of Genetics and Genomic Sciences, Icahn School of Medicine at Mount Sinai, New York City, NY 10029, USA; boymin2010@163.com

**Keywords:** competing endogenous RNA, long non-coding RNA, regulatory network, WGCNA, differentially expressed RNAs, thyroid carcinoma

## Abstract

RNAs may act as competing endogenous RNAs (ceRNAs), a critical mechanism in determining gene expression regulations in many cancers. However, the roles of ceRNAs in thyroid carcinoma remains elusive. In this study, we have developed a novel pipeline called Molecular Network-based Identification of ceRNA (MNIceRNA) to identify ceRNAs in thyroid carcinoma. MNIceRNA first constructs micro RNA (miRNA)–messenger RNA (mRNA)long non-coding RNA (lncRNA) networks from miRcode database and weighted correlation network analysis (WGCNA), based on which to identify key drivers of differentially expressed RNAs between normal and tumor samples. It then infers ceRNAs of the identified key drivers using the long non-coding competing endogenous database (lnCeDB). We applied the pipeline into The Cancer Genome Atlas (TCGA) thyroid carcinoma data. As a result, 598 lncRNAs, 1025 mRNAs, and 90 microRNA (miRNAs) were inferred to be differentially expressed between normal and thyroid cancer samples. We then obtained eight key driver miRNAs, among which hsa-mir-221 and hsa-mir-222 were key driver RNAs identified by both miRNA–mRNA–lncRNA and WGCNA network. In addition, hsa-mir-375 was inferred to be significant for patients’ survival with 34 associated ceRNAs, among which *RUNX2*, *DUSP6* and *SEMA3D* are known oncogenes regulating cellular proliferation and differentiation in thyroid cancer. These ceRNAs are critical in revealing the secrets behind thyroid cancer progression and may serve as future therapeutic biomarkers.

## 1. Introduction

Thyroid cancer is the most common malignancy of endocrine organs with enormous heterogeneity in terms of morphological features and prognosis [1]. Although most thyroid carcinomas tend to be biologically indolent and have a good prognosis, there are a few associated with more aggressive clinical manifestations [2]. Thyroid carcinoma is popularly classified into four classes including anaplastic thyroid carcinoma (ATC), follicular thyroid carcinoma (FTC), medullary thyroid carcinoma (MTC), and papillary thyroid carcinoma (PTC) [3], among which PTC is most common [4,5]. In 2017, there are approximately 56,870 new thyroid cancer incidences, representing about 3.4% of all new cancer cases worldwide [6]. Similarly, in the United States, the incidences of thyroid carcinoma increased steadily by around 6.6% each year from 2002 to 2009, which is the highest among all cancers [7]. In fact, thyroid cancer has become the fifth most common cancer in women [7,8]. 

Even though thyroid cancer harbors several highly universal genetic alterations, some of which are unique to this cancer [3], it is still very challenging to predict this cancer due to the complex disease progression process and complicated molecular interactions involved in it. Over the past decades, numerous studies have been performed to predict this cancer based on molecular, morphological, and immunological features [9], most of which focused on the detection of cancer-related protein-coding genes. However, it is known that protein-coding RNAs only cover approximately 2% of the total transcripts in mammalian [10], which urges the need to study the functions of non-coding RNAs (ncRNAs), especially long ncRNAs (lncRNAs) [11]. 

Recently, significant progresses have been achieved in exploring lncRNA biology [12]. For example, the lncRNA *PVT1* was shown to be up-regulated in PTC, FTC, and ATC; *MALAT1* was inferred to be involved in the regulation of cell cycle and migration [13]. However, most studies only focus on a subset of lncRNAs with specific regulatory mechanisms, while less is known on a transcriptome wide scale [14]. Moreover, the interaction mechanism between many kinds of RNAs are still elusive. Recent studies suggest that RNAs regulate each other’s expression levels by competing for a limited pool of microRNAs (miRNAs) in some circumstances [15,16]. In particular, Poliseno and his colleagues proposed a hypothesis: there exists an intricate post-transcriptional regulatory network mediated by miRNAs, in which non-coding RNAs and protein-coding RNAs compete for binding to miRNAs and regulate each other’s expression via sharing one or more miRNA response elements (MREs) [15]. The ncRNAs and protein-coding RNAs are called competing endogenous RNAs (ceRNAs) in the hypothesis. 

ceRNA hypothesis demonstrates a new level of post-transcriptional regulation [17]. Given that even complete relief from repression by a miRNA usually has only mild effects on an individual mRNA, this theory highlights the importance of sharing binding sites for different miRNAs to yield substantial crosstalk [18,19,20]. ceRNAs may play a major role in certain dis-regulated or transient cellular states. For instance, it has been shown that the expression of tumor-suppressor gene *PTEN* can be regulated by its miRNA-mediated competitors *VAPA*, *CNOT6L*, *SERINC1* or *ZNF460* [21]. Especially, such mechanisms seem to be of particular relevance in cancer. For example, the lncRNA linc-MD1 has been shown to regulate the skeletal muscle cell differentiation clock by sponging miRNAs from its competitors, thereby enacting a ceRNA mechanism. In this ceRNA mechanism, *MAML1* and *MEF2C* compete with linc-MD1 for miR-133 and miR-135, respectively [22,23]. Nevertheless, it is very difficult to build the exact ceRNA network and use it to understand RNA competing mechanisms. Fortunately, there are many well-established RNA databases such as long-non-coding RNA-associated diseases (LncRNADisease) database [24], the Human miRNA Disease Database (HMDD) [25], and database of Differentially Expressed MiRNAs in human Cancers dbDEMC [26]. In addition, miRNA-target interaction databases including miRcode [27] and miRanda [28,29,30,31,32,33,34,35], and ceRNA databases such as long non-coding competing endogenous database (lnCeDB) [36] have been developed, which provide much useful information.

In this study, we develop a novel pipeline called Molecular Network-based Identification of ceRNA (MNIceRNA) to identify ceRNAs in thyroid carcinoma. MNIceRNA first performs differential RNA analyses using edgeR [37], and then constructs gene co-expression and regulatory networks using machine learning based methods such as weighted correlation network analysis WGCNA [38] and known interaction data downloaded from databases such as miRcode. Finally, MNIceRNA focuses on thyroid carcinoma associated key driver genes (KDGs) and constructs a ceRNA network according to the lnCeDB [36]. The functions of the identified KDGs are also explored.

## 2. Materials and Methods 

### 2.1. Data Collection and Pre-Processing

We downloaded RNA expression profiles of thyroid cancer and control samples from the Genomic Data Commons (GDC) data portal [39,40] and patient’s clinical information (see Table 1) from The Cancer Genome Atlas (TCGA) database [39,40]. Specifically, there are 559 samples used in this study, including 501 primary tumor samples and 58 solid tissue normal samples. The Genome research project of ENCyclopedia of DNA Elements (GENCODE) (GRCh38) (v25) catalogue (http://www.gencodegenes.org/) was used as a reference to quantify lncRNAs and mRNAs. In summary, 15,540 lncRNAs and 19,848 mRNAs from RNA-Sequencing (RNA-Seq) and 1881 miRNAs from miRNA-Seq were retrieved.

### 2.2. Differential Gene Expression Analysis

We applied edgeR to identify differentially expressed RNAs [37]. Specifically, the gene read counts were first processed with one scaling normalized factor from Trimmed Mean of M values (TMM) [41]. The negative binomial (NB) model was then applied to calculate the significance of RNAs, followed by an adjustment of *p*-values using the Benjamini–Hochberg method [42]. The cut-off values for significantly expressed RNAs were: (1) the false discovery rate (FDR, the adjusted *p* value) < 0.001; and (2) |log_2_ fold change (FC)| > 2 [5].

### 2.3. Construction of Gene Regulatory Network

We reconstructed the regulatory network using data combining lncRNAs, mRNAs, and miRNAs. The lncRNA–miRNA interactions and miRNA–mRNA interactions were downloaded from miRcode [27]. We then adopted a software called key driver analysis (KDA) [43] to identity key drivers in the regulatory network. Specifically, KDA takes a set of genes G and a directed gene network N as inputs. In our study, G is the differentially expressed RNAs and N is the regulatory network [44].

### 2.4. Construction of Gene Co-Expression Network

We inferred the co-expression network for a set of 32,209 RNAs including lncRNA, miRNA and mRNA using weighted gene co-expression network (WGCNA) algorithm [45], which was then visualized by Cytoscape 3.4.0 [12,46].

### 2.5. Survival Analysis

Survival analysis was performed using Cox proportional hazards regression models, with RNA expression in samples established as a binary variable. Because patient’s age and tumor stage have been interpreted to deeply influence molecular traits and clinical effect in thyroid cancer, we limited our initial cohort to primary tumor >Stage I patients. Specifically, we performed survival analyses for 9 (8 miRNA from miRcode, and 1 miRNA from WGCNA) miRNA, 80 lncRNA, and 190 mRNA (Table S1). *p*-values generated under this model were corrected for multiple-hypothesis testing using the Benjamini–Hochberg correction, with a significance threshold of FDR < 0.05 [47].

### 2.6. Function Enrichment

For clustering analysis of the significantly expressed RNAs, a pairwise complete-linkage hierarchical clustering method was employed to calculate the Euclidean distance. The results were shown using a heat map generated from the software packages cluster 3.0 [48] and TreeView [49]. In addition, differentially expressed genes (DEGs) were annotated by the Database for Annotation, Visualization and Integrated Discovery (DAVID) tool (V6.8) [50,51], and Kyoto Encyclopedia of Genes and Genomes (KEGG) analysis was also used to discover the potential pathways involved [12].

## 3. Results

### 3.1. Differentially Expressed RNAs between Primary Tumor and Control Samples

**Figure 1:**
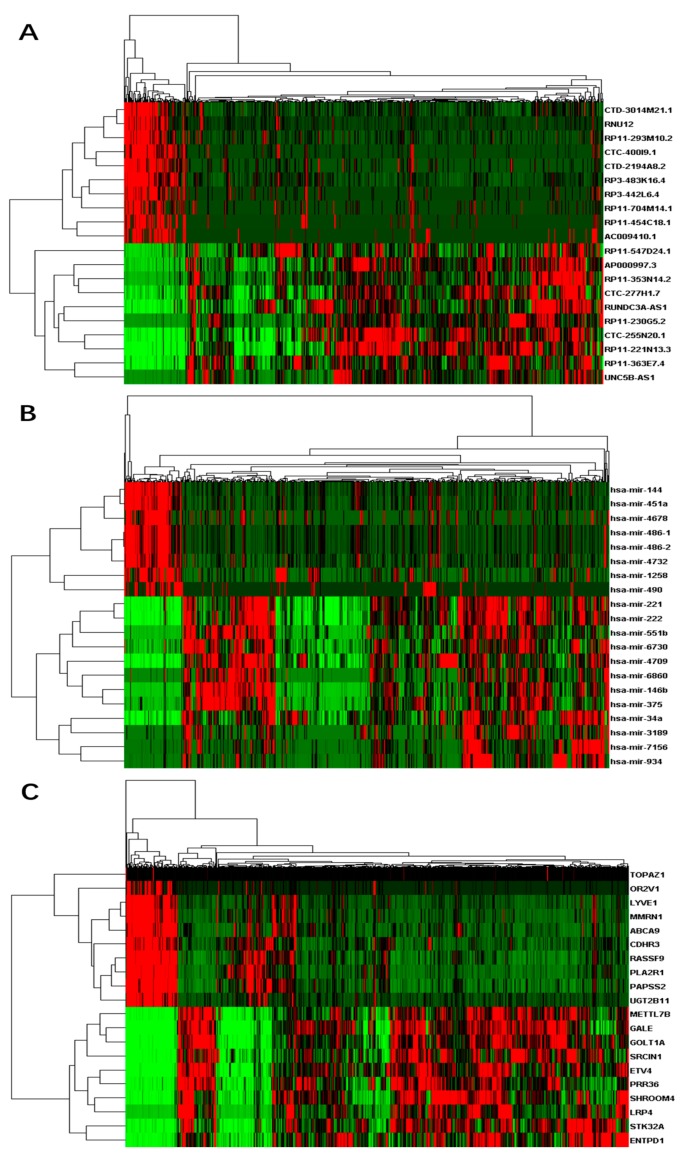
Heat-maps on expressions of top differential RNAs for thyroid cancer: (**A**) differential long non-coding RNAs (lncRNAs); (**B**) differential micro RNAs (miRNAs); and (**C**) differential messenger RNAs (mRNAs). *X*-axis represents samples, while *Y*-axis represents the biological elements studied.

A total of 60,488 genes from RNA-Seq including 15,540 (26%) lncRNAs and 19,848 (33%) mRNAs were detected according to GENCODE (GRCh38) (v25) annotation. We also obtained 1881 miRNAs from miRNA-Seq. Similar to the genotype-tissue expression (GTEx) study [52], we performed a few data processing steps to require that genes to have at least 0.1 fragments per kilobase million (FPKM) in 2 or more individuals followed by quantile normalization across genes. In total, 14,848 lncRNAs, 16,575 mRNAs, and 786 miRNAs were left after the filtering. Based on the differential analyses, we inferred 463 up-regulated differentially expressed lncRNAs and 135 down-regulated differential lncRNAs, 812 up-regulated differential mRNAs and 213 down-regulated differential mRNAs, and 82 up-regulated differential miRNAs and eight down-regulated miRNAs respectively (Table S2). We selected a few top differential RNAs in each category and plotted their expression heat-maps in Figure 1.

### 3.2. Enriched Functions of Differentially Expressed RNAs

**Figure 2:**
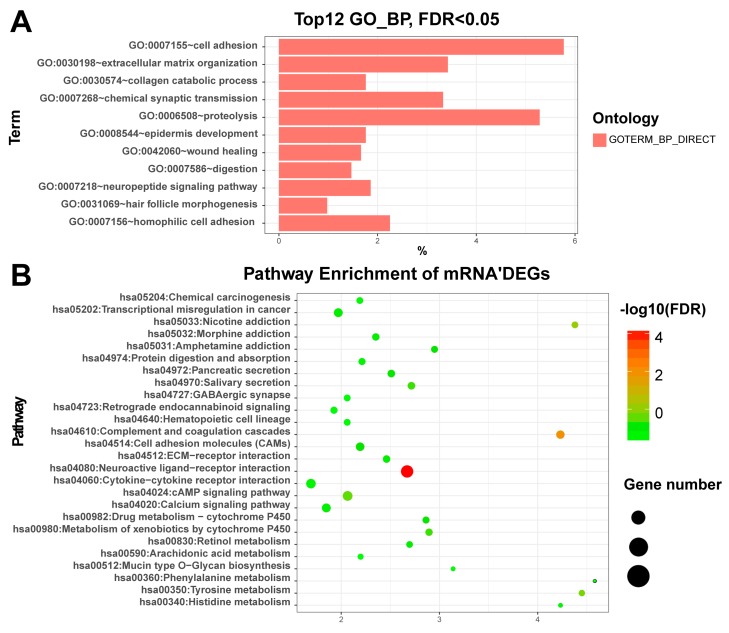
Plot of the differentially expressed genes enriched GO and KEGG: (**A**) The plot of the enriched GO (biological process) with DEGs of the mRNAs. *X*-axis represents the percentage of enriched genes. (**B**) The plot of the enriched KEGG with DEGs of the mRNAs. GO: Gene Ontology; KEGG: Kyoto Encyclopedia of Genes and Genomes; DEGs: Differentially expressed genes; FDR: False discovery rate. miRNAs: The differentially expressed miRNAs were significantly enriched in miRNAs in cancer (hsa05206, FDR = $4.44\times 10^{-14}$), which include hsa-mir-221, hsa-mir-222, hsa-mir-31, hsa-mir-34a, hsa-mir-373, hsa-mir-375, hsa-mir-451a, hsa-mir-483, hsa-mir-520a, hsa-mir-520c, hsa-mir-520g, and hsa-mir-520h. Among them, Wu et al. [54] found that miR-31 was significantly down-regulated in papillary thyroid carcinoma patients. Furthermore, down regulation of miR-31 increased the proliferation, migration, and invasion of ovarian carcinoma cells. In addition, they revealed that the human antigen R (HuR) was a target for miR-31 and knock down of HuR resulted in enhanced cell viability and decreased cell migration rate.

mRNAs: The functional enrichment analysis revealed that up-regulated DEGs were significantly enriched in the synthesis and degradation of extracellular matrix (ECM) related terms such as ECM organization (GO:0030198, FDR = $7.76\times 10^{-7}$) and ECM disassembly (GO:0022617, FDR = 0.05), collagen catabolic process (GO:0030574, FDR = $2.85\times 10^{-5}$), and cell adhesion (GO:0007155, FDR = $4.3\times 10^{-7}$) (Table S3). Based on the pathway enrichment analysis, the over-represented pathways of up-regulated DEGs was Neuroactive ligand-receptor interaction (hsa04080, FDR = $7.05\times 10^{-5}$) (Table S4). We also plotted the top enriched gene ontology (GO) and KEGG terms in Figure 2 for a better view. Our results are basically in line with Qiu et al. [5], especially ECM–receptor interaction, activation of MAPK activity and positive regulation of MAPK cascades [53].

lncRNAs: There are only 3 differentially expressed lnRNAs annotated in GO, namely RP11-161M6.2, ELFN2 and LINC00473. ELFN2 and LINC00473 were assigned to GO term “negative regulation of phosphatase activity (GO:0010923)” and “transcription, DNA-templated (GO:0006351)”, respectively. RP11-161M6.2, also called lipase maturation factor 1 (LMF1), played significant roles in many GO terms including triglyceride metabolic process (GO:0006641), endoplasmic reticulum (ER) to Golgi vesicle-mediated transport (GO:0006888), protein secretion (GO:0009306), protein glycosylation in Golgi (GO:0033578), chylomicron remnant clearance (GO:0034382), lipid digestion (GO:0044241), positive regulation of lipoprotein lipase activity (GO:0051006), protein maturation (GO:0051604), regulation of cholesterol metabolic process (GO:0090181), and regulation of triglyceride metabolic process (GO:0090207). In addition, there is only one lncRNA MIR205HG annotated in KEGG pathway “MiRNAs in cancer (hsa05206)”.

### 3.3. Key Driver Analysis

We have miRNA targets on 79,940 lncRNAs and 375,324 mRNAs and a total of 455,264 interaction pairs were collected from miRcode. We called this regulatory network the Database Network. We then mapped DEGs onto the Database Network, and performed key driver analysis to infer key genes driving the DEGs. As a result, we identified eight key driver miRNAs: hsa-mir-507, hsa-mir-375, hsa-mir-31, hsa-mir-144, hsa-mir-221, hsa-mir-222, hsa-mir-184, and hsa-mir-187. As annotated in DAVID database, hsa-mir-375, hsa-mir-31, hsa-mir-221, hsa-mir-222 and hsa-mir-184 were miRNAs in cancer, among which hsa-mir-184 was also relevant to endothelial dystrophy-iris hypoplasia-congenital cataract-stromal thinning (EDICT) syndrome. Interestingly, Zhang et al. [13] found that hsa-mir-375 [55] was up-regulated in PTC and MTC; hsa-mir-144 was up-regulated in PTC; hsa-mir-187 was up-regulated in PTC and FTC; and hsa-mir-221 [56,57] and hsa-mir-222 [56,58] were up-regulated in PTC, FTC, and ATC [55], which confirm the intimate association of our key drivers to thyroid cancer. 

To study the influence of network structure to MNIceRNA, we reconstructed the co-expression network for the set of 32,209 RNAs using WGCNA. Sixty-five modules were identified, among which blue module is contains the largest number of DEGs. These genes of module were enriched in oxidation-reduction process and thyroid hormone synthesis. We then used this network to infer key driver genes and acquired 273 key drivers, including three miRNAs, 190 mRNAs and 80 lncRNAs, among which two (out of three) miRNAs, hsa-mir-221 and hsa-mir-222, overlapped with previous key driver miRNAs. The miR221-222 cluster is located on the X chromosome. Many previous studies have shown that the miR221-222 cluster was in the downstream of the MAPK pathway and involved in the regulation of cell cycle and apoptosis [13]. They were well known for deregulation in various malignancies and were among the first group of miRNAs shown to be deregulated in thyroid carcinoma [58,59]. In terms of mechanism, it was shown that miR221-222 cluster plays functions in PTC through negatively regulating p27 [60] and in ATC through their interaction with p27, RECK, and PTEN [59]. Similar other malignancies, up-regulation of miR221-222 cluster was associated with increased treatment resistance and recurrence rate, worse prognosis, and more invasive disease course [59,61,62]. It was likely that their tumorigenic property in thyroid was associated with their functions in tumor invasion and epithelial-mesenchymal transition (EMT) as shown in other malignancies [61,63]. They also act as potential biomarkers of thyroid malignancy with worse prognosis.

### 3.4. Competing Endogenous RNA Network Reveals Competing Endogenous Mechanisms of Long Non-Coding RNAs and Messenger RNAs

**Figure 3:**
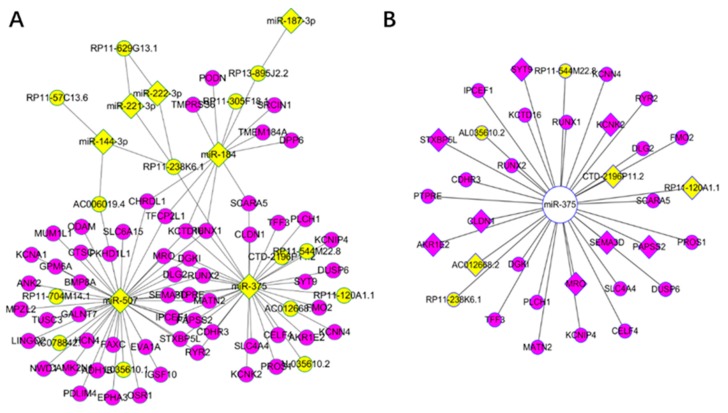
Key drivers for Thyroid carcinoma in the long non-coding competing endogenous database (lnCeDB): (**A**) yellow nodes represent miRNAs (diamond) and lncRNAs (ellipse), while the purple represent mRNAs; and (**B**) yellow nodes represent lncRNAs, purple represent mRNAs, and diamond represent significant RNAs in the survival analysis from key driver analysis (KDA) of the weighted correlation network analysis (WGCNA). The above relationship are competing endogenous (ceRNAs) from the lnCeDB database.

We focused on KDGs and constructed a ceRNA network according to lnCeDB database. As a result, 97 ceRNAs were obtained, most of which were also KDGs identified using the WGCNA network. Specifically, there were 34 ceRNAs related to hsa-mir-375, among which 11 were significant in the survival analysis (Figure 3). As examples, *RUNX2* and *SEMA3D* are known oncogenes [5] and *DUSP6* is critical for PTC and the MAPK pathway [64,65].

### 3.5. Survival Analysis of Key Driver Genes

**Figure 4:**
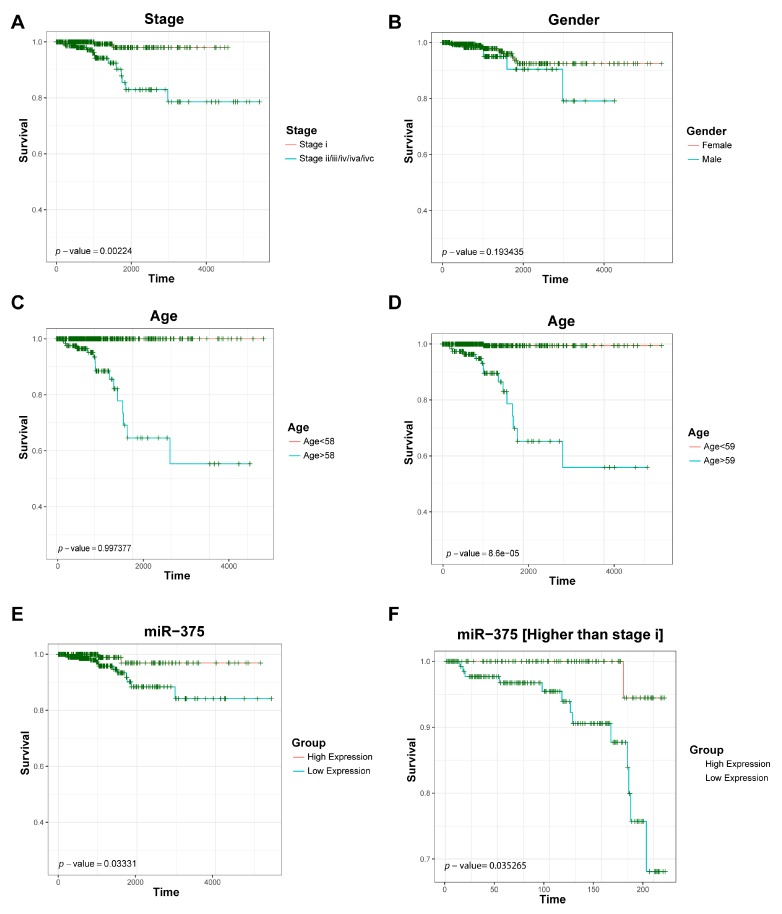
Thyroid patients’ survival analyses on miR-375 and a few clinical traits: (**A**,**B**) survival outcomes of different stages and gender; (**C**,**D**) different age groups have different effects on over-all survival; and (**E**,**F**) survival outcomes according to relatively high and low expression, of which (**E**) represents survival outcomes of has-mir-375 in all samples, and (**F**) represents survival outcomes of has-mir-375 in higher than stage I tumor.

We studied the association of key driver RNAs with patients’ survival, which can be used to evaluate their prognostic potential, and plotted in Figure 4 the Kaplan–Meier overall survival curves for miR-375 and a few clinical traits including cancer stage, gender and age. As a result, Figure 4A shows that cancer stage is significantly associated with overall survival with a *p*-value of 0.002, while gender and age (59 and above) were not significantly associated. Interestingly, Figure 4E,F shows that miR-375 is significantly associated to survival (*p*-value 0.03), indicating that it might be a prognostic maker and drug target for thyroid cancer. In addition, we also identified 44 significant mRNAs and 13 significant lncRNAs (*p* value < 0.05) in the survival analyses. Among them, lncRNAs RP5-1024C24.1 and LINC01539 were verified to be differentially expressed in PTC and exhibit specific topological characteristics in the lncRNA–mRNA co-expression network [66]. LncRNA RP5-1024C24.1 is an antisense transcript of metallophosphoesterase domain containing 2 (*MPPED2*). A previous study has shown that *MPPED2* functions as a tumor suppresser in neuroblastoma tumorigenesis, and thus the low expression of RP5-1024C24.1 might promote tumor progression [66]. In addition, LINC01539 are deregulated in PTC [13].

## 4. Discussion

MNIceRNA combines information of RNA co-expression, regulation, and competing endogenous mechanisms to identify potential thyroid cancer biomarkers. It infers highly meaningful biomarkers as validated by literature mining and survival analyses. Specifically, MNIceRNA identified a cohort of 463 up-regulated differential lncRNAs, 812 differential mRNAs, and 82 differential miRNAs, which were mainly enriched in the ECM organization and degradation pathway. Similarly, there were 135 down-regulated differential lncRNAs, 213 down-regulated differential mRNAs, and eight down-regulated differential miRNAs, mainly enriched in cancer (hsa05206). Five up-regulated mRNAs, namely *ELF3*, *HMGA2*, *LCN2*, *MET*, and *RUNX2*, are known oncogenes [5]. In addition, as the up-regulated differential RNA exhibiting the most TF activity, *PLAU* encodes a serine protease that acts as an activator in the ECM degradation in tumor development [67]. *PLAU* also plays crucial roles in tumor invasion and metastasis [68]. Its overexpression has been found in cancer associated fibroblasts, which contribute to the tumor growth and progression [69]. Recently, a few studies suggested that *PLAU* was induced by PTC [70]. In contrast, a set of down-regulated mRNAs such as *EGR2*, *GPC3*, *IGFBPL1*, *LRP1B*, *NR4A3*, and *PROX1* were identified as TSGs [5]. As a vital TF exhibiting higher transcriptional activity in PTC samples than in control, *EGR2* functions as a tumor suppressor that is generally decreased in numerous cancer types such as ovarian [71] and gastric cancer [72]. These collectively suggest that *PLAU* might server as a significant TF promoting PTC progression, while *EGR2* might be a tumor suppressor. However, more experimental validations are needed [5]. As for lncRNA, AC007255.8 is an antisense transcript of proline rich 15 (*PRR15*), which is overexpressed in advanced stage human colorectal cancer [73] and its expression was correlated with patient age [66]. AC079630.2 is mostly enriched in the pathway “Transcriptional misregulation in cancer”. Luo et al. showed that AC079630.2 exhibited high diagnostic ability to distinguish normal tissue and PTC tissue [74]. HOX transcript antisense RNA (*HOTAIR*) has been shown to be deregulated in a great number of human cancers such as oral cancer, nasopharynx, breast, esophagus, lung, liver, pancreas, colon, endometrium and cervix [75]. A study based on the TCGA and Gene Expression Omnibus (GEO) showed that HOTAIR was associated with poor survival of thyroid cancer patients [76]. 

In addition, the KDA analysis revealed eight key miRNAs, hsa-mir-375/507/222/221/187/184/144/31, among which hsa-mir-375 was significantly associated with patients’ survival. Previous studies suggested that overexpression of miR-375 was frequently observed in thyroid cancer patients and thus it might play a critical oncogenic role and be a potential diagnostic biomarker in thyroid cancer [77,78,79,80,81]. For example, the expression level of miR-375 was comparable between primary tumors and matched lymph node metastases, suggesting that its expression pattern in nodal metastases could prominently reflect that of the primary tumor in MTC [81]. Lassalle et al. found that the up-regulation of miR-375 was accompanied by reduced cell growth and synergistically improved sensitivity to vandetanib [80]. They also observed that miR-375 could enhance *PARP* cleavage and decline AKT phosphorylation, which play vital roles in cell growth. In addition, Wang et al. indicated that overexpression of miR-375 could inhibit the PTC cells proliferation and this inhibition was caused by the induction of cell apoptosis [82]. Moreover, it was found that miR-375 was upregulated by more than 35-fold in Follicular Variant Papillary Thyroid Carcinoma (FVPTC) and 20-fold in classic PTC, and not upregulated in normal thyroid tissue, hyperplastic nodules, and follicular carcinomas [83]. Thus, hsa-mir-375 is associated with early- and late-stage malignant progression and might be a novel clinical biomarker for thyroid cancer. 

Moreover, using the lnCeDB database, MNIceRNA identified 34 ceRNAs (including six lncRNAs and 28 mRNAs) associated with hsa-mir-375, among which 11 were significant in the survival analysis. As examples, microarray analysis revealed that hypothyroidism induces significant reductions in *KCNK2* transcripts [84]. *CLDN1* is an essential molecule in tight junctions, *CLDN1* was highly expressed in normal thyroid epithelium, but reduced in Hashimoto's Thyroiditis (HT) injured thyroid epithelial cells [85]. In addition, *SEMA3D* had superior diagnostic accuracy independently of the cytology in six datasets including The Cancer Genome Atlas (TCGA) thyroid dataset. This gene exhibited differences in the correlation coefficients between benign and malignant samples and could be an effective clinical biomarker for diagnosis of thyroid cancer [86]. Similarly, Steffen et al. [87] assembled a miRNA–protein target network for 677 human miRNAs and 18,880 targets which are listed in the TargetScan (http://www.targetscan.org). In this list, we found that hsa-mir-507 has a corresponding protein complex, corum-1422, and hsa-mir-221-222 cluster regulates nine protein complexes, of which *ITGB1* [88], *ITGB3* [88] and *CD47* [89] were associated with the thyroid cancer. However, none of these protein complexes were putative ceRNAs we predicted.

It is worth mentioning that, although MNIceRNA identified many literature- and experiment-validated miRNAs, lncRNAs and mRNAs associated with thyroid cancer, the extent and relevance of ceRNA effect in vivo is still poorly understood. Recent experimental studies have suggested that the miRNA-mediated competition between ceRNAs could constitute an additional level of posttranscriptional regulation, playing important roles in many biological contexts. The sensitivity analysis shows that binding free energy and repression mechanisms are key ingredients for cross-talk between ceRNAs to arise. Interactions emerging in specific ranges of repression values, can be symmetrical (one ceRNA influences another and vice versa) or asymmetrical (one ceRNA influences another but not the reverse), and may be highly selective, while possibly be limited by noise [90]. On the other hand, the reporter assays demonstrated that [91] only active miRNA families with low total miRNA:target ratios are susceptible to ceRNA inductions even up to approximately 10,000 additional target copies per cell. In summary, there were many criteria for validating ceRNA, such as quantitative measurements of miRNA and target abundance [92], miRNA concentration and the size and affinities of the competing target pool [91], miRNA:target ratio, the absolute concentration of the effective target pool [91], timescales, steady-state, kinetic parameters [23,93], cellular concentrations of RBPs and miRNAs [20], and so on. In addition, PTR model was introduced in Figliuzzi et al. [90,93] by characterizing the transient response of the system to perturbations to validate ceRNA. The method was divided into two parts. The first part focuses on small perturbations by analyzing (in Fourier space) the linearized dynamics of a system of N ceRNAs jointly targeted by a single miRNA species. The second part focuses instead on large perturbations by using numerical analysis and analytical estimations to characterize the emergence of nonlinear response. The PTR model can be used to analyze the stoichiometric relationship of miR-375 and its target sites by manipulating TA through controlled expression of a validated target of miR-375 in thyroid [92]. Finally, the titration mechanism [93], argonaute individual-nucleotide resolution cross-linking and immune-precipitation (iCLIP) [91], Hermes systematically [94], stochastic model [95], Gillespie algorithm [95,96] and dynamical method [93] could also be used to validate ceRNAs. In the future, we will adopt these methods and perform experiments to validate the ceRNAs we predicted. 

Taking together, our study identified many literature-validated RNAs critical to thyroid cancer progression and proposed a few novel RNAs to function as competing endogenous RNAs for thyroid cancer. However, we are fully aware that the limited sample size and information on miRNA–lncRNA–mRNA interactions might restrict the power of our conclusions. More experimental validations are suggested to confirm the contribution of our proposed RNAs in thyroid cancer. 

## 5. Conclusions

In summary, we proposed a more multifaceted approach to construct ceRNAs network, and identified a set of crucial genes that could be used as biomarkers for thyroid carcinoma therapy, such as hsa-mir-375, AC012668.2, and *SEMA3D*. In the future, more attention should be paid to the construction of ceRNA networks and the validation of biomarkers or RNA competing endogenous interactions.

## Figures and Tables

**Table 1:** Clinical information of the 559 samples used in this study.

Characteristic		Numbers
Sample type	Primary tumor	501
	Solid tissue normal	58
Age	Median	47
	Range [years]	15~89
Sex	Male	152
	Female	407
Vital status	Alive	539
	Dead	20
Stage	I	315
	II	59
	III	124
	IV	2

## Supplementary Materials

The following are available online at www.mdpi.com/2073-4425/9/1/44/s1. Table S1: Survival analysis of key driver RNAs, Table S2: Up- and down-regulated differential RNAs, Table S3: Function enrichment of differential expressed RNAs on Gene ontology biological processes, Table S4: Function enrichment of differential expressed RNAs on KEGG pathways.
